# Quantification of Aquaporin-Z reconstituted into vesicles for biomimetic membrane fabrication

**DOI:** 10.1038/s41598-017-11723-x

**Published:** 2017-09-14

**Authors:** Hui Xian Gan, Hu Zhou, Qingsong Lin, Yen Wah Tong

**Affiliations:** 1 National University of Singapore, NUS Environmental Research Institute (NERI), Singapore, 117411 Singapore; 2 National University of Singapore, Chemical and Biomolecular Engineering, Singapore, 117576 Singapore; 3 National University of Singapore, Department of Biological Sciences, Singapore, 117543 Singapore

## Abstract

Aquaporin incorporated biomimetic membranes are anticipated to offer unprecedented desalination capabilities. However, the lack of accurate methods to quantify the reconstituted aquaporin presents a huge hurdle in investigating aquaporin performance and optimizing membrane fabrication. Herein, we present three quantification methods to determine the Aquaporin-Z reconstituted into *E. coli* lipid vesicles: 1) nanogold labeling with transmission electron microscopy (TEM) visualization, 2) nickel labeling with inductively coupled plasma-mass spectrometry (ICP-MS) and 3) gel electrophoresis. The TEM method serves as a quick way to determine if aquaporin has been reconstituted, but is not quantitative. The numerical results from quantitative methods, ICP-MS and gel electrophoresis, correlate closely, showing that 60 ± 20% vs 66 ± 4% of Aquaporin-Z added is successfully reconstituted into vesicles respectively. These methods allow more accurate determination of Aquaporin-Z reconstituted and loss during reconstitution, with relatively commonly available equipment and without complex sample handling, or lengthy data analysis. These would allow them to be widely applicable to scientific studies of protein function in the biomimetic environment and engineering studies on biomimetic membrane fabrication.

## Introduction

Reverse osmosis is the leading technology in water desalination. The burgeoning need for water has fueled the search for higher performance membrane that can reduce the energy consumption and cost of reverse osmosis process^1^. Aquaporin (Aqp) water channel proteins provide passage to more than three billion water molecules per second per molecule while maintaining rejection to other solutes in cell membranes^2, 3^. It is anticipated that Aqp-reconstituted biomimetic membrane has the greatest potential for performance enhancement but yet furthest from commercial viability^4, 5^. While many fabrication strategies and lab scale testing has been presented over the years, few fundamental studies have been done in this area and most works remain rudimentary^6–18^.

Aqp is the main component that distinguishes a biomimetic membrane from a conventional membrane, yet the actual amount of Aqp reconstituted is often neglected due to the lack of quantification methods. This hinders systematic understanding of the intricate works of the biomimetic membrane, which is vital for scientific exploration and membrane performance optimization. Theoretically, a larger number of Aqp incorporated into vesicles or membrane should result in improved membrane performance. However, several studies have reported that increasing protein to lipid reconstitution ratio beyond a critical point led to deteriorated vesicle permeability instead^8, 12, 13, 15, 18^. Unexpectedly, there are also reports of poorer membrane performance with immobilization of vesicles of greater permeability^10, 12^. Nonetheless, the root cause of such phenomenon cannot be pinpointed without proper quantification methods.

Currently, stopped-flow light scattering (SFLS) serves as a standard method to evaluate Aqp reconstitution^19^. SFLS works as a functional assay, whereby protein incorporated vesicles are mixed rapidly with a solution of high osmolarity, which results in water efflux from vesicles. During the course, the rate of shrinkage of vesicles is measured to determine vesicle permeability. However, this method is an indirect representation of Aqp reconstitution since permeability can also be affected by a plethora of other factors such as vesicle quality, incorporation matrix, Aqp conformation and orientation in the bilayer. Furthermore, the stopped-flow assay is vulnerable to large signal to noise ratio for a sample of poor quality^19, 20^. There are several challenges in developing accurate quantification methods, including interferences from incorporation matrix or non-reconstituted Aqp. Additionally, the quantification method should also satisfy numerous criteria for it to be practical and useful. These include requiring small sample consumption, using relatively commonly available equipment and involving no complicated sample handling or data processing processes.

There has been an attempt to answer the need for quantification of Aqp reconstituted by the Danish company Aquaporin A/S^19^. They have presented several quantification methods including freeze-fracture transmission electron microscopy (FF-TEM), fluorescence correlation spectroscopy (FCS) and small-angle X-ray scattering (SAXS)^19^. However, FF-TEM can give ambiguous results for polymeric systems. Conversely, the latter two methods can provide detailed information but require access to large-scale facilities and knowledge in modelling and fitting for data progressing.

**Figure 1:**
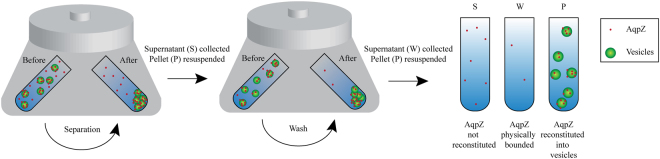
Ultracentrifugation to separate non-reconstituted AqpZ from AqpZ reconstituted in vesicles.

In this paper, we introduce three accurate and quantitative methods to determine the Aquaporin-Z (AqpZ) successfully reconstituted into *Escherichia coli* (*E. coli*) lipid vesicles. In order to enable accurate quantification, it is important to remove interferences by separating the non-reconstituted AqpZ from the vesicles prior to further analysis. This is achieved through ultracentrifugation of samples such that the reconstituted AqpZ was pelleted down along with vesicles while non-reconstituted AqpZ remained in the supernatant (Fig. 1). These three different fractions obtained, supernatant (S), wash (W) and pellet (P), are then subjected to further analysis using the three quantification methods which would be discussed in the following section.

We aim to present quantification methods that can be performed with relatively commonly available equipment, without complicated sample handling or data processing. The three methods are namely: (1) nanogold labeling and transmission electron microscope (TEM) visualization, (2) nickel labeling and inductively coupled plasma-mass spectroscopy (ICP-MS) quantification and (3) gel electrophoresis. The advantages and disadvantages of each method will be further discussed. Based on the accuracy required and equipment availability, a suitable quantification method can be selected for the application. Conversely, these methods can also be applied in parallel for verification of results and harnessing more information.

## Results and Discussion

### Stopped-flow light scattering permeability test

**Figure 2:**
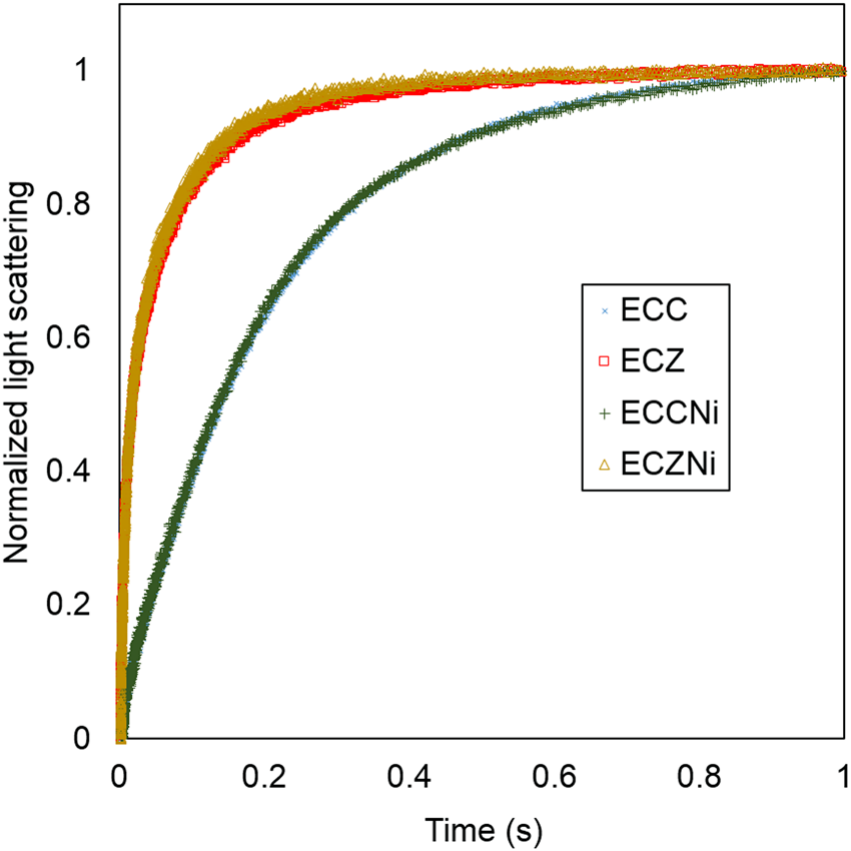
Stop-flow light scattering curve. An average of at least 5 independent curves is shown for each. ECC and ECZ refer to unlabeled control and AqpZ reconstituted *E. coli* lipid vesicles respectively. ECCNi and ECZNi refer to nickel labeled control and AqpZ reconstituted *E. coli* lipid vesicles respectively.

AqpZ-incorporated *E. coli* lipid vesicles were employed to demonstrate the viability of the three proposed protein quantification methods in this work. Control vesicles were prepared by adding a buffer without AqpZ during reconstitution. The effectiveness of AqpZ reconstitution was first evaluated with conventional methods, SFLS permeability test and dynamic light scattering (DLS). Successful AqpZ incorporation is evident from the results of SFLS functional assay (Fig. 2 and Table 1), which shows a marked increase in vesicle permeability from 24 µm/s to 800 µm/s with AqpZ addition. DLS results in Table 1 also show that vesicles of around 170 nm in diameter were formed for both control and AqpZ incorporated vesicles. However, it is also clear from the results that SFLS method is only able to provide information about vesicle permeability, but not further information on number and distribution of Aqp reconstituted in vesicles.

**Table 1:** Diameter of vesicles measured by DLS, size distribution by intensity.

	Diameter (nm)	Polydispersity index (PDI)	Kinetic rate constant, k (1/s)	Permeability, P_f_ (µm/s)
ECC	170 ± 10	0.08 ± 0.01	4.5 ± 0.6	24 ± 2
ECZ	166 ± 8	0.10 ± 0.02	150 ± 40	800 ± 200
ECCNi	170 ± 10	0.10 ± 0.01	4.4 ± 0.8	23 ± 3
ECZNi	170 ± 8	0.12 ± 0.02	130 ± 20	700 ± 90

Note: The kinetic rate constant is determined from curve fitting on 5 to 10 stopped-flow measurements. Standard deviation is calculated from results obtained from three independent repeated experiments. ECCNi and ECZNi refer to nickel labeled control and AqpZ reconstituted *E. coli* lipid vesicles respectively. ECC and ECZ refer to unlabeled control and AqpZ reconstituted *E. coli* lipid vesicles respectively.

A critical evaluation of Aqp performance or reconstitution methods and materials can only be conducted with complete information on both actual Aqp incorporation and P_f_, as shown for the calculation of P_a_ in equation (6). While the increase in P_f_ value does demonstrate the function of Aqp, it is only useful for comparison between control and Aqp reconstituted vesicles prepared under same experimental conditions. It is insufficient for understanding AqpZ performance or effectiveness of reconstitution under varying incorporating conditions. A fair assessment of AqpZ performance will require normalization of P_f_ values with vesicle dimensions and the actual number of AqpZ reconstituted in vesicles.

Other than the use for accurate evaluation of Aqp performance, actual Aqp reconstitution is also an essential information for engineering biomimetic desalination membranes. Vesicle immobilization is a preferred strategy for producing Aqp biomimetic desalination membrane^19^. Accordingly, immobilization of vesicles with highest P_f_ attainable has been the main pursuit of many work^8, 12, 13, 18^. There has been a wide range of vesicle size reported, ranging from a diameter of 80 to 200 nm, varying with method and material for reconstitution^7, 10, 15–17^. Notably, vesicles of larger size would likely be able to accommodate a greater number of AqpZ per vesicle and achieve a larger P_f_ value. Yet, vesicles of higher P_f_ may not necessarily result in better membrane performance as larger vesicle sizes would limit the number of vesicles that could be immobilized on the membrane^20^. In all, optimization of membrane performance would require information on vesicle dimensions and actual AqpZ reconstituted, on top of P_f_.

### Validation of ultracentrifugation separation method

To quantify the incorporated protein in vesicles, it is vital to remove the non-reconstituted AqpZ from the vesicles prior to further analysis. The effectiveness of the ultracentrifugation separation method was validated through separation of bovine serum albumin (BSA) from *E. coli* vesicles. BSA was selected as the control protein due to its inability to be incorporated onto the vesicle membrane bilayer of *E. coli* vesicle. Due to the water-soluble nature of BSA, it should be distributed predominantly in the buffer, with a limited amount bound on surface of vesicles. As such, an effective separation method will allow the BSA doped into *E. coli* vesicle solution to be predominantly recovered in the supernatant. However, we would like to highlight the limitations of BSA as a control as it is not a membrane protein and may behave differently from AqpZ in its interaction with lipid membrane and separation under ultracentrifugation. The choice of BSA as a control is due to the lack of a better alternative protein which behaves similarly to AqpZ and yet gets incorporated into the bilayer.

BSA was doped in *E. coli* control vesicles at a lipid to protein weight ratio of 100 and mixed well at 750 rpm for 2 h. Ultracentrifugation was then performed twice to ensure complete separation of non-reconstituted BSA, including those that are just physically bound onto the vesicles. The procedure is as shown in Fig. 1, with the exception of having BSA in place of AqpZ. The three fractions collected were analyzed with gel electrophoresis. BSA can be identified from its band position corresponding to around 55 kDa and quantified by comparison against a calibration curve constructed from a series of a known amount of BSA.

**Figure 3:**
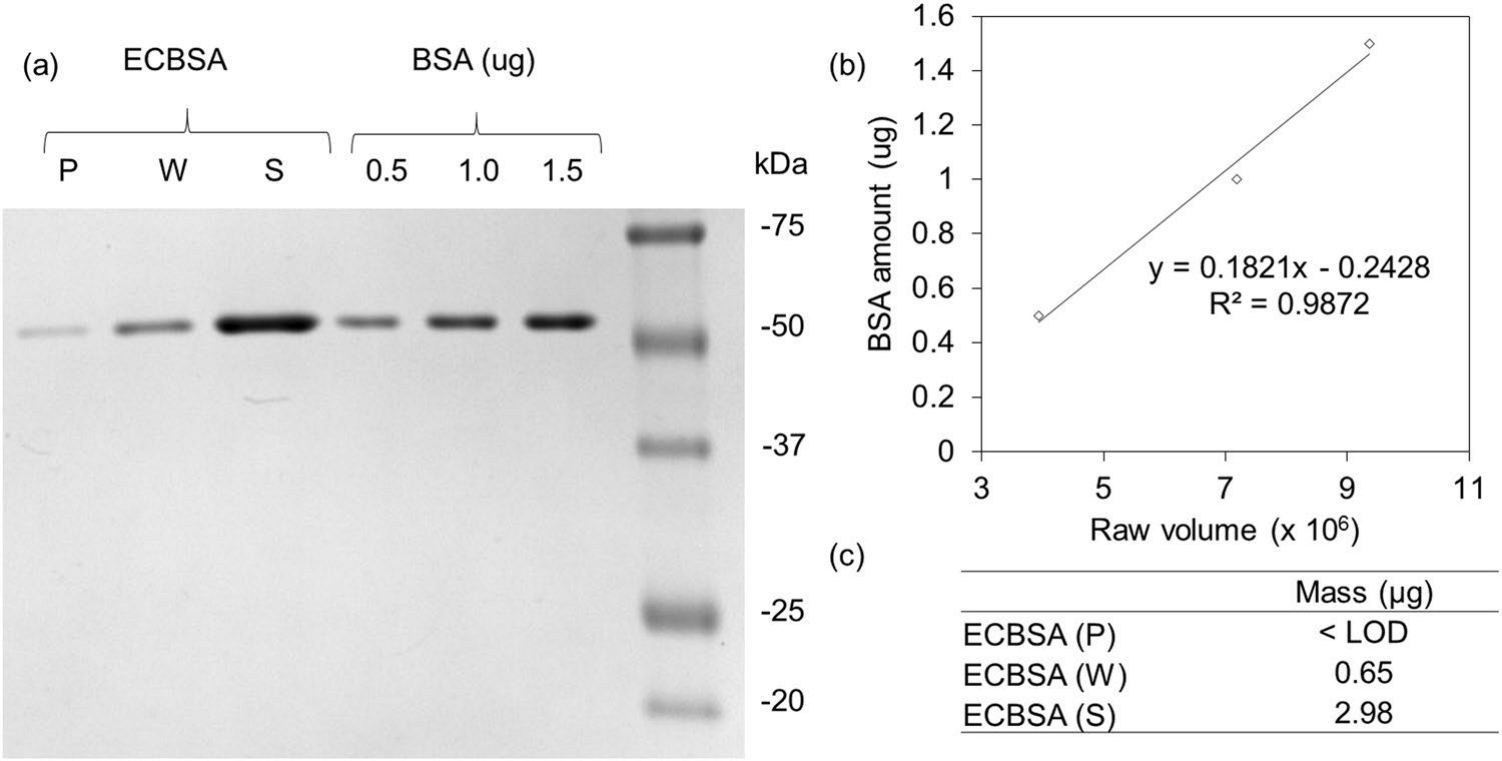
Gel electrophoresis results of different fractions of BSA-doped *E. coli* vesicles. (**a**) Gel electrophoresis result, the full length gel is presented in Supplementary Figure S1. (**b**) BSA calibration curve constructed from loading known mass BSA of 0.5 µg, 1.0 µg and 1.5 µg for gel electrophoresis. (**c**) Mass of BSA present in different fractions, determined through BSA calibration curve. ECBSA refers to BSA-doped *E. coli* control sample. P, W and S refer to the pellet, wash and supernatant fraction obtained from after ultracentrifugation. BSA refers to BSA standard solution loaded in known mass as indicated.

The results shown in Fig. 3 demonstrate the effectiveness of separation of non-reconstituted BSA predominantly into the supernatant and wash fraction. There is a total of 4.00 µg of BSA loaded in the BSA doped *E. coli* vesicles, out of which, 2.98 µg was detected in the supernatant fraction and 0.65 µg was found in the wash fraction. After ultracentrifugation, a small amount of BSA that is beyond the quantification limit of our working curve can be observed in the pellet fraction (Fig. 3a). This could be attributed to BSA which is absorbed onto the vesicles and hence remains in the pellet fraction. Notably, the total mass of BSA recovered in three fractions sum up to 90.1% of the BSA doped initially, with the other 10% of BSA either lost during the ultracentrifugation process or remaining in the pellet fraction.

Since the ultracentrifugation procedure was shown to effectively separate non-reconstituted protein from vesicles, the same procedure was applied to AqpZ reconstituted vesicles and control vesicles (with and without nickel labeling). This procedure yielded three different fractions for each sample, supernatant (S), wash (W) and pellet (P), as shown in Fig. 1. The bulk of the non-reconstituted AqpZ will remain in supernatant from the first round of ultracentrifugation. Conversely, minute amount AqpZ which could be physically bound to the surface of vesicles can be separated via resuspension and a second round of ultracentrifugation in the wash fraction. The measured amount of pelleted AqpZ is likely to be the maximum amount of AqpZ that is functionally integrated into the membrane since the pellet may also contain non-functional integrated AqpZ that are aggregated, trapped or absorbed onto vesicles.

### Nanogold labeling and TEM visualization

TEM is commonly employed to confirm vesicle formation, yet it is difficult to distinguish reconstituted Aqp unambiguously from the collapsed polymer chains of vesicles under TEM^19^. We propose to enhance the information that can be gathered using TEM by labeling His-tagged AqpZ with Ni-NTA linked nanogold. These 5 nm spherical nanogold, with a distinct size and shape and high electronic conductivity, can be easily visualized under TEM to determine the success of AqpZ reconstitution on vesicles. This would allow information about the morphology of vesicle and distribution of AqpZ on vesicles to be obtained simultaneously. Nickel ions recognize and bind to histidine tag (His-tag) with high affinity, as its imidazole ring offers electron donor groups that coordinate with the metal ions^21, 22^. This interaction was leveraged to conduct the labeling. The on-grid labeling procedure was optimized to minimize non-specific binding and performed as described in the experimental section.

**Figure 4:**
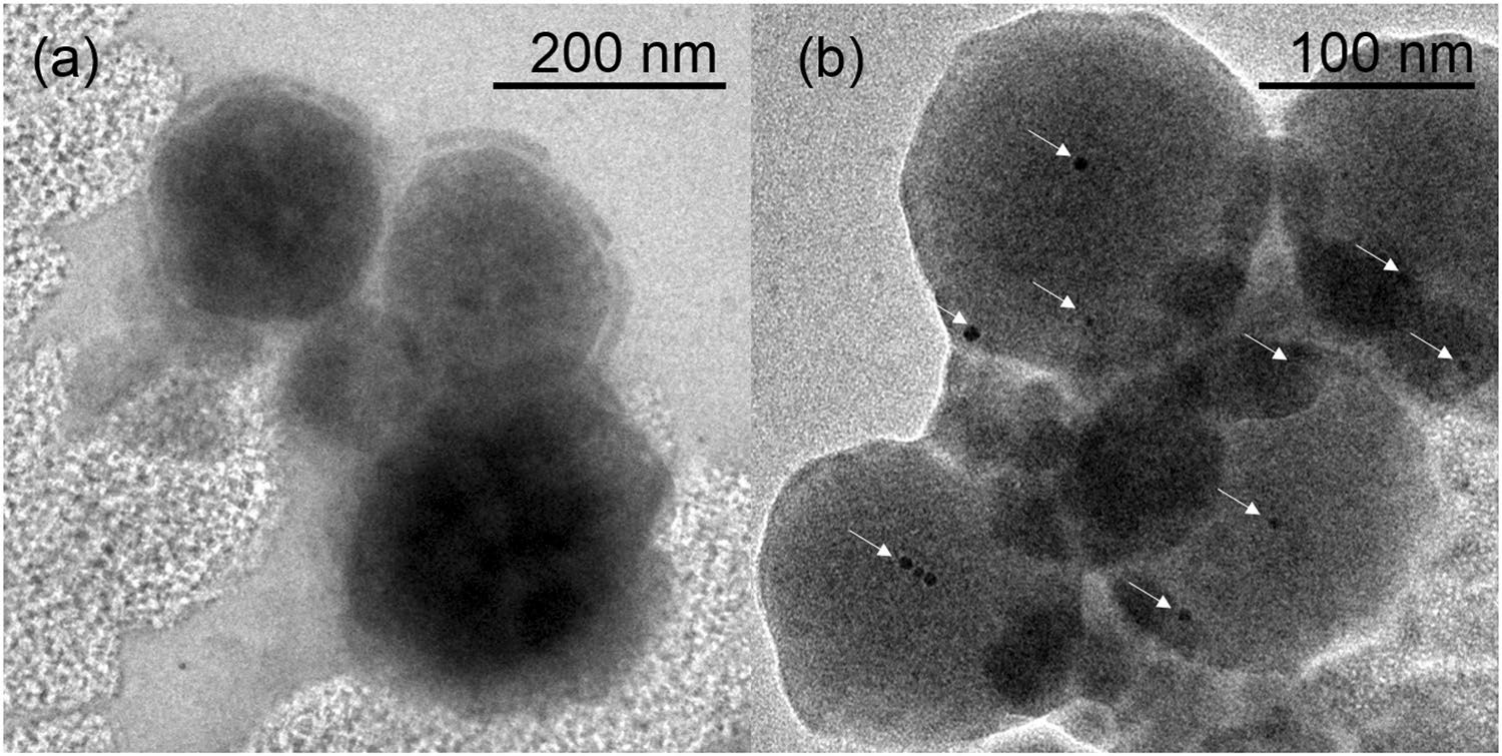
Nanogold labeling TEM results. (**a**) Gold-labeled *E. coli* control vesicles without AqpZ. (**b**) Gold-labeled *E. coli* vesicles with AqpZ reconstituted at a lipid to AqpZ weight ratio of 100. White arrows point towards 5 nm Ni-NTA nanogold.

The TEM images show that control (without protein) does not have nanogold bound while those with AqpZ shows nanogold bound (Fig. 4). In this case, the TEM images show 2 to 3 nanogold bound on each vesicle. This method serves as a convenient method for quick visualization of Aqp reconstituted into vesicles. The labeling and TEM experiment only takes 2 h to complete. Furthermore, TEM visualization is a necessary characterization technique to determine vesicle formation. An additional step of nanogold labeling allows reconstitution information to also be obtained. This method is exceptionally useful for a quick screening of different materials for vesicle preparation and incorporation method. Nonetheless, it should be applied with the caution that the result does not hold statistical significance until larger effort and time is devoted to counting the number of nanogold on a huge population of vesicles.

The nanogold number observed could be an underestimation of the actual number of AqpZ reconstituted. Since the 5 nm nanogold is similar in size to AqpZ and can cause steric hindrance to reconstitution, labeling has to be performed after reconstitution step. As such, His-tags on AqpZ which are oriented towards the inner core of vesicles will be inaccessible for nanogold labeling and the actual amount of AqpZ incorporated can be underestimated. In addition, the large size of nanogold would hinder binding to His-tag in close proximity, which would be the case for His-tags on the same tetramer. Lastly, the vesicles may cluster together during TEM sample preparation, posing greater difficulties in distinguishing the nanogold on each different vesicle. Since we are unable to measure the channel function after labeling the AqpZ with nanogold, no comparison of channel activity with other methods or literature can be performed. The aforementioned reasons limit the value of TEM method for accurate quantification and led us to develop the next two approaches for more quantitative measurements.

### Nickel labeling and ICP-MS quantification

In order to circumvent the limitation of nanogold labeling method, we propose to label AqpZ with nickel (II) ions, which are of picometre size and are significantly smaller compared to AqpZ^23^. This allows labeling to be performed before reconstitution without affecting the incorporation and AqpZ function. Based on two-tailed *t*-test, there is an insignificant difference in the diameter between ECZ and ECZNi (P > 0.05) and between their ECC and ECCNi (P > 0.05). Similarly, *t*-test shows an insignificant difference in P_f_ between ECZ and ECZNi (P > 0.05) and between their ECC and ECCNi (P > 0.05).

#### Determination of binding molar ratio between nickel and His-tag

Nickel labeled AqpZ reconstituted can easily be quantified by measuring the nickel concentration, as long as the binding stoichiometric relation between nickel and His-tagged Aqp can be determined^21^. As such, verification of the binding ratio between nickel and His-tag is crucial for this method. For that, AqpZ and control buffer were labeled with nickel chloride solution in large excess and unbound nickel was removed by a desalting column and dialysis. The samples were then subjected to microwave digestion and ICP-MS. The protocol was optimized such that it allowed complete removal of nickel from the control buffer without AqpZ. Briefly, the nickel labeling amount, method of nickel removal, MWCO of dialysis cassette were investigated. Eventually, the dialysis buffer was collected for analysis to check that nickel concentration is reduced to a negligible amount in dialysis buffer after three buffer replacements. (Please refer to the supporting information for data on optimization.)

**Figure 5:**
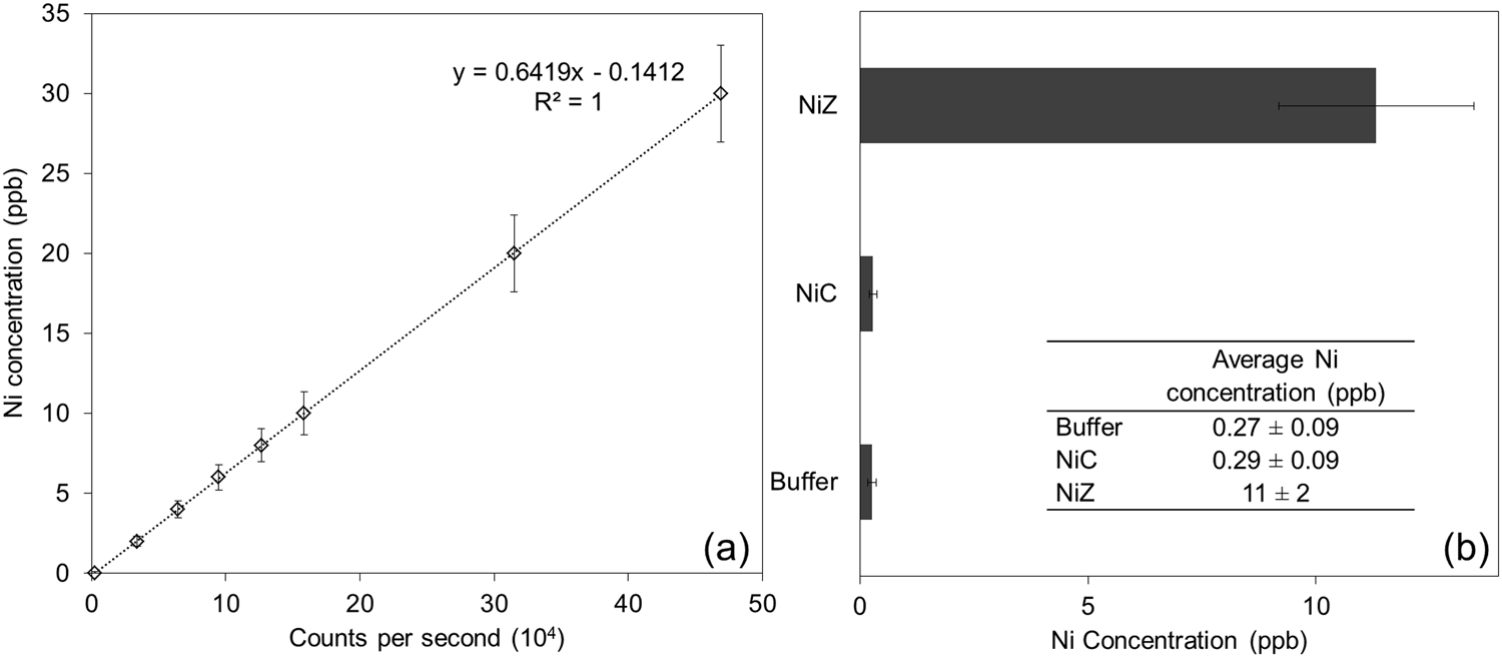
ICP-MS results of nickel labeled AqpZ. (**a**) Representative calibration curve for ICP-MS measurement. Standard deviation is calculated from results obtained from three independent repeated experiments. (**b**) Labeling process was performed three times and nickel concentration was determined from ICP-MS. Standard deviation is calculated from results obtained from three independent repeated experiments. Buffer refers to 20 mM Tris-HCl, 100 mM NaCl buffer with 0.2% DDM. NiC and NiZ refer to nickel labeled control and AqpZ respectively. 8 mg/L of AqpZ or equivalent volume of control buffer which were microwave digested was used for ICP-MS.

ICP-MS is a highly sensitive technique to investigate trace metal concentration in samples^24^. This makes ICP-MS a suitable method to detect the nickel concentration in our samples. The results from nickel labeled AqpZ are presented in Fig. 5. The calibration curve shows that the nickel measurement is done within the linear range of readings. Based on the measured nickel concentration and the concentration of AqpZ determined using RCDC (reducing agent and detergent compatible) assay (Bio-Rad), the binding molar ratio between histidine and nickel is 1.8. This result is consistent with other published work that pH above 5 favors the behavior of His-tag as a tridentate ligand that coordinates with nickel ions in an octahedral geometry, giving rise to bis-histidine-nickel complex^21^. The dissociation constant for the interaction between His-tag and Ni^2+^ was reported to be around 10^−6^ M at neutral pH, showing the high stability of this interaction^25^. With a known binding ratio, AqpZ amount per unit volume of vesicle sample can be calculated with the measurement of nickel concentration from incorporated AqpZ with nickel labeling.

#### Quantification of AqpZ based on nickel concentration

**Figure 6:**
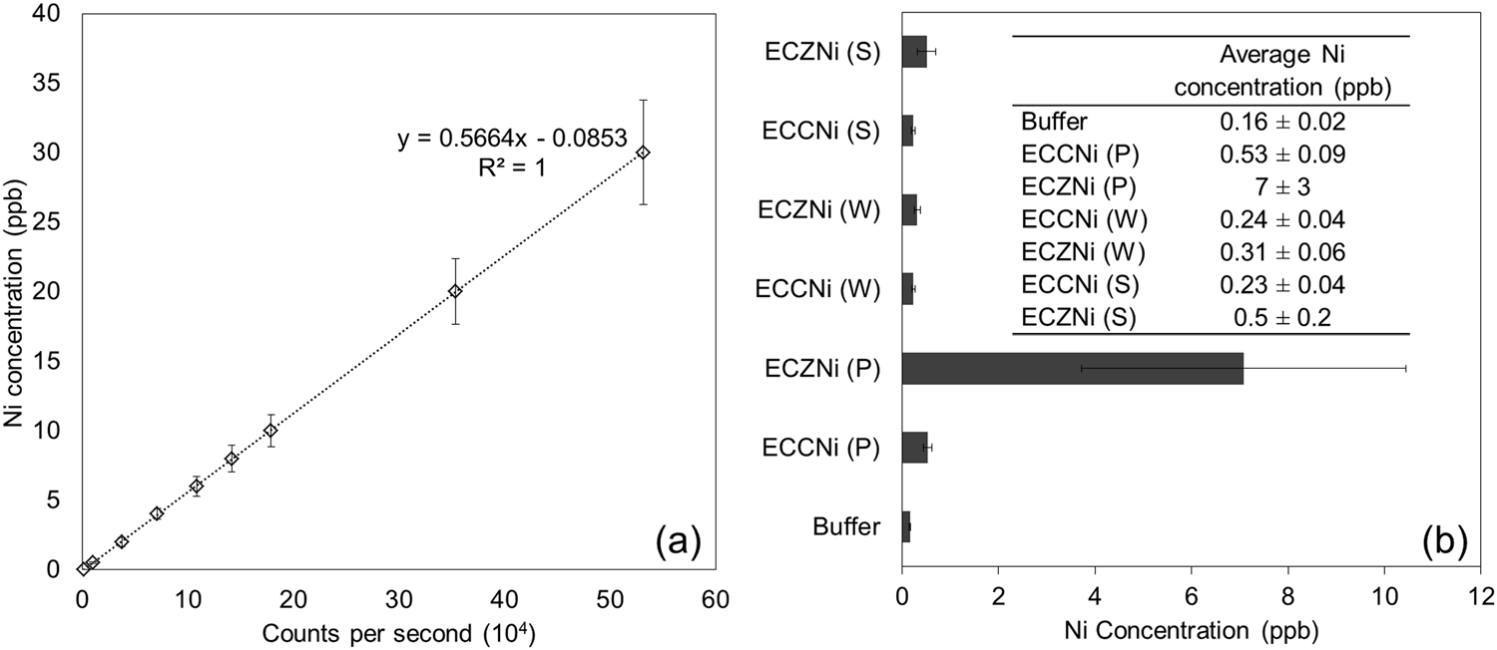
ICP-MS results of nickel labeled AqpZ incorporated into vesicles. (**a**) Representative calibration curve for ICP-MS measurement. Standard deviation is calculated from results obtained from three independent repeated experiments. (**b**) Reconstitution process was performed three times and nickel concentration was determined from ICP-MS. Buffer refers to 0.1 M MOPS buffer each sample was suspended in. Standard deviation is calculated from results obtained from three independent repeated experiments. ECCNi and ECZNi refer to nickel labeled control and AqpZ reconstituted *E. coli* lipid vesicles respectively. P, W, S refers to the pellet, wash and supernatant fractions collected after ultracentrifugation. 8 mg/L of AqpZ or an equivalent volume of control and buffer which are microwave digested is used for ICP-MS.

Next, this nickel labeled AqpZ or control buffer were reconstituted into *E. coli* lipid vesicles. A portion of each sample was subjected to ultracentrifugation before ICP-MS quantification analysis. Samples were broken down via microwave digestion before injecting into ICP-MS for quantification. The reconstitution and quantification procedure was performed three times. Results from the reconstitution of nickel labeled into *E. coli* lipid vesicles are summarized in Fig. 6.

#### Percentage of AqpZ successfully reconstituted

The percentage of AqpZ successfully reconstituted, with respect to total amount of AqpZ added, can be calculated using equation (1).

1 $$ \% \,{\rm{AqpZ}}\,{\rm{reconstituted}}\,=\,\frac{{\rm{Reading}}\,{\rm{for}}\,{\rm{pellet}}}{{\rm{Reading}}\,{\rm{before}}\,{\rm{dialysis}}}\times 100 \% $$

Percentage of AqpZ in pellet fraction indicates the proportion of added AqpZ which were successfully reconstituted into *E. coli* vesicles, the rest of AqpZ were either lost through the incorporation process or not reconstituted. Using our current method of incorporation, 60 ± 20% of the AqpZ added in the beginning was reconstituted successfully.

#### Identification of procedure undermining AqpZ reconstitution

Besides the percentage of AqpZ added that was successfully reconstituted, ICP-MS results can also be used to identify specific steps that lower incorporation. With that, specific conditions can be identified for optimization to improve reconstitution. The concentration of AqpZ at different stages of the reconstitution experiment can be monitored by collecting and measuring their nickel concentrations. In this work, the nickel concentration of samples collected at three different stages of the experiment, namely, before reconstitution dialysis, after reconstitution dialysis, and after ultracentrifugation was measured.

Each reading for AqpZ incorporated *E. coli* was corrected for the dilution effects of dialysis and deducting the reading of corresponding control samples before using for calculation. The resulting nickel concentration of AqpZ reconstituted *E. coli* vesicles at each stage of incorporation are tabulated in Table 2. It is evident that nickel concentration, which is directly proportional to AqpZ concentration, decreases after reconstitution dialysis and decreases even further after ultracentrifugation.

**Table 2:** Tabulation of ICP-MS results of nickel labeled AqpZ reconstituted *E. coli* samples (ECZNi) at different stages in the experiment.

ECZNi	Corrected nickel concentration (ppb)
Before dialysis	10 ± 2
After dialysis	8 ± 3
After ultracentrifugation (P + W + S)	7 ± 4
P	7 ± 3
W	0.08 ± 0.03
S	0.3 ± 0.2

Note: 0.8 mL of each sample (at 10 mg/mL *E. coli* lipid concentration) was loaded for ICP-MS analysis. Standard deviation is calculated from results obtained from three independent repeated experiments. The nickel reading after ultracentrifugation separation is determined from the summation of readings for all three fractions (pellet, wash, and supernatant) of a sample. P, W, S refers to pellet, wash and supernatant fractions respectively.

Since the dilution effect of dialysis has been accounted for, the remaining loss in nickel concentration can be attributed to AqpZ loss during the reconstitution and ultracentrifugation process. Using equations (2), (3) and (4), the percentage of AqpZ loss through dialysis and ultracentrifugation or AqpZ which are not lost in the reconstitution process but yet not reconstituted into vesicles, can be calculated from the corrected nickel concentration (Table 3). 20 ± 10% of AqpZ was lost during the dialysis process, this is significantly higher than the 3.4 ± 0.9% of AqpZ remaining not reconstituted in the solution. This means that AqpZ which are not lost are predominantly reconstituted successfully into the vesicles. This further indicates that the primary reason for reduced efficiency of this reconstitution method is the AqpZ loss during incorporation, such as due to adherence onto dialysis membrane or loss to external dialysis buffer due to defects in dialysis membrane, rather than the lack of capacity for vesicles to accommodate more AqpZ.

2 $$ \% \,{\rm{AqpZ}}\,{\rm{loss}}\,(\mathrm{dialysis})\,=\,\frac{{\rm{Before}}\,{\rm{dialysis}}\,-\,{\rm{After}}\,{\rm{dialysis}}}{{\rm{Before}}\,{\rm{dialysis}}}\times 100 \% $$

3 $$ \% \,{\rm{AqpZ}}\,{\rm{loss}}\,(\mathrm{ultracentrifugation})\,=\,\frac{{\rm{After}}\,{\rm{dialysis}}\,-\,({\rm{P}}+{\rm{W}}+{\rm{S}})}{{\rm{Before}}\,{\rm{dialysis}}}\times 100 \% $$

4 $$ \% \,{\rm{AqpZ}}\,{\rm{not}}\,{\rm{reconstituted}}\,=\,\frac{{\rm{W}}\,+\,{\rm{S}}}{{\rm{Before}}\,{\rm{dialysis}}}\times 100 \% $$

**Table 3:** Calculated percentage of AqpZ loss, not reconstituted or reconstituted, based on results from ICP-MS and gel electrophoresis.

	ICP-MS	Gel electrophoresis	
	ECZNi	ECZNi	ECZ
% AqpZ loss (Dialysis)	20 ± 10	9 ± 8	8 ± 16
% AqpZ loss (Ultracentrifugation)	15 ± 4	20 ± 10	26 ± 14
% AqpZ not reconstituted	3.4 ± 0.9	<LOD	<LOD
% AqpZ reconstituted	60 ± 20	72 ± 8	66 ± 4

Note: Standard deviation was derived from measurements obtained from three independent reconstitution and quantification experiments. <LOD refers to below limit of detection.

#### Calculation of permeability per AqpZ monomer (P_a_)

The combination of vesicle permeation measurement by SF, dimension test by DLS, and protein incorporation amount determination can be applied to investigate the performance of single protein. Since the lipid to protein weight ratio added is known to be 100, the actual amount of AqpZ reconstituted and the average AqpZ number per vesicle can be calculated. The permeability per AqpZ monomer (P_a_) can be calculated from P_f_ and vesicle molecular weight using the method previously reported^3, 26^. At 100% incorporation, there would be an average of 52.7 tetramers per vesicle for a lipid to AqpZ weight ratio of 100. With the known actual percentage of incorporated AqpZ of 60 ± 20%, the average number of tetramer per vesicle is 32 ± 9. Coupled with stopped-flow data presented for nickel labeled samples previously, P_a_ was calculated to be 51 × 10^−14^ cm^3^/s/subunit. With 95% confidence, the average P_a_ is between 20 × 10^−14^ cm^3^/s/subunit to 80 × 10^−14^ cm^3^/s/subunit. As expected, the value determined is higher than those reported in the literature, whereby P_a_ was reported to be greater than 10 × 10^−14^ cm^3^/s/subunit^3^ and 32.4 × 10^−14^ cm^3^/s/subunit^17^, as the incorporation was assumed to be 100% for those experimental studies Conversely, a P_a_ value of 16 ± 5 cm^3^/s/subunit has been reported for molecular dynamics simulations done on AqpZ^27^. This shows the relevance of quantification methods in critical evaluation of AqpZ performance.

Nickel labeled AqpZ and ICP-MS is a highly accurate and sensitive quantification method to determine AqpZ reconstituted into vesicles. This is evident from its ability to detect the small percentage of AqpZ which is not reconstituted. The great sensitivity allows this quantification to be useful for both scientific studies on AqpZ interactions with the environment and optimization of biomimetic membrane fabrication. However, due to the additional steps involved in labeling AqpZ and measuring the nickel binding ratio prior to reconstitution, this method requires a longer duration of up to 2 weeks to complete. Additionally, the number of lipid vesicles was calculated assuming a negligible loss. Furthermore, nickel dissociating from AqpZ during sample handling and transfer cannot be verified for this method and may contribute to error in protein estimation.

### Gel electrophoresis

Unlike the previous two quantitation methods which rely on the detection of labels interacting with His-tag, gel electrophoresis is applied to measure protein amount by detecting a dye-stained protein band in the gel. Therefore, gel electrophoresis is able to provide a direct read out of protein amount rather than indirect information depending upon the label. During gel electrophoresis, incorporated AqpZ in vesicles is extracted by SDS detergent into gel sample loading buffer. The identity of AqpZ can then be determined by comparing its band position against a protein marker ladder and the corresponding amount can be further deduced by comparison with a BSA standard working curve.

**Figure 7:**
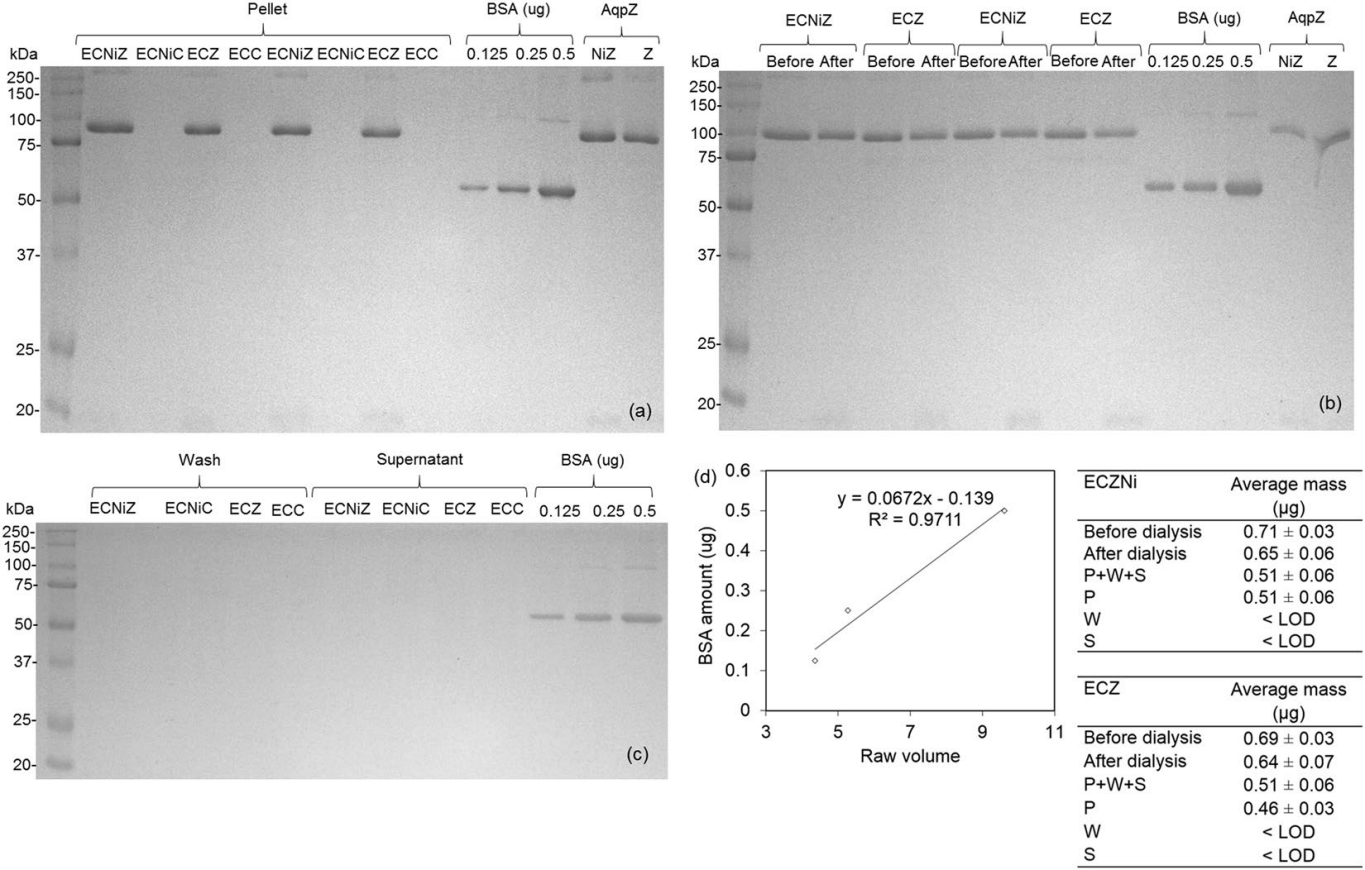
Gel electrophoresis results of AqpZ incorporated vesicles. (**a**–**c**) Electrophoretic analysis of *E. coli* lipid samples before and after incorporation via dialysis, full length gels are presented in Supplementary Figure S2(a–c). Samples were derived from the same experiment and the gels were processed using same settings in parallel. Sample containing 1 ug of AqpZ or equivalent volume of control buffer is loaded for gel electrophoresis. Duplicates of each sample were loaded for each gel electrophoresis for (**a**–**b**). Standard deviation is calculated from results obtained from three independent repeated experiments. Before and after refer to before and after dialysis respectively. Pellet, wash, and supernatant refers to the different fractions collected from ultracentrifugation separation process. ECC and ECZ refer to control and AqpZ reconstituted *E. coli* lipid vesicles respectively. ECCNi and ECZNi refer to nickel labeled control and AqpZ reconstituted *E. coli* lipid vesicles respectively. NiZ refers to nickel labeled AqpZ and Z refers to non-nickel labeled AqpZ. (**d**) BSA standard working curve and quantified AqpZ results.

The incorporation and quantification procedure was performed three times using unlabeled AqpZ and nickel labeled AqpZ. The representative gel result is shown in Fig. 7(a–c). As expected, only samples with AqpZ added for reconstitution shows a band which corresponds to the same position as AqpZ protein, at around 90 kDa. Since control samples do not have AqpZ added, the band at 90 kDa is absent for all control fractions. This confirms the identity of the band as a representation of AqpZ, which can be used to determine AqpZ amount in each sample.

#### Quantification of AqpZ based on AqpZ amount

To quantify the protein in different fractions, Coomassie blue staining method was applied using BSA as the standard, as previously reported^28^. A series of BSA standard, at various dilutions, was prepared to obtain a calibration curve within a linear range to quantify the AqpZ amount based on band densitometry (Fig. 7(d)). The Coomassie blue staining method is based on a quantitative binding of Coomassie blue dye (Coomassie brilliant blue G250) to proteins. AqpZ is known to maintain its tetrameric state on Coomassie blue stained gels but dissociates into monomers and dimers on SDS-PAGE gels^29^. Hence, Coomassie blue stained gel is selected for quantification of tetrameric AqpZ incorporated in this work. Theoretically, the amino acid composition and structural difference of different proteins vary the dye binding to the protein, and therefore the working curve of this method^30^. Therefore, we would like to highlight that BSA, a non-membrane protein, may exhibit a difference in staining by Coomassie blue as compared to AqpZ. This can contribute to error in protein estimation due to the working curve. Despite the inherent differences that limit the value of BSA as a control, it is difficult to find a better control protein. Since BSA has been adopted as a standard protein for AqpZ quantification in other work^28, 31^, BSA is selected as the standard in our work. Using the GelScan software for analysis, the amount of AqpZ in each fraction of the sample was obtained. The average result obtained from three independent reconstitution and quantification experiments are shown in Fig. 7(d). Although there is no need for AqpZ to be labeled with Ni ions, gel electrophoresis method was applied to ECZNi for a comparison with the ICP-MS method. It is observed that ECZNi has consistently higher reading than ECZ in the Coomassie blue staining method. This could be due to the presence of nickel in ECZNi compared to ECZ. Since Ni carries a positive charge, it can result in extra binding of the negatively charged dye to the sample, which is then evaluated as a larger amount of protein. Since the method is meant to be applied for non-labeled AqpZ, we will focus on the results obtained from ECZ in the following sections.

#### Percentage of AqpZ successfully reconstituted

The previous equations (1) to (4) used for evaluating AqpZ percentage reconstitution and loss can also be applied here, with the exception of using AqpZ amount reading instead of nickel concentration reading. Using equation (1), the percentage of AqpZ reconstituted with respect to the total amount of AqpZ added for incorporation is 66 ± 4%. This is comparable to the percentage of reconstituted AqpZ evaluated using the ICP-MS results, 60 ± 20%.

#### Identification of procedure undermining AqpZ reconstitution

In this case, there was no band at the 90 kDa position for both supernatant and wash fraction for AqpZ incorporated vesicle samples, showing that AqpZ is predominantly reconstituted. Similar to ICP-MS results, a large proportion of AqpZ appears to be lost through dialysis and ultracentrifugation processes.

Nonetheless, it should be noted that there could still be a minute amount of AqpZ which exist in the wash and supernatant fraction, but yet too low to be detected under gel electrophoresis. This can be cross verified with results from ICP-MS, which shows 3.4% of AqpZ are not lost during the incorporation process but yet not successfully reconstituted into vesicles. The method may also suffer from errors in protein estimation due to loss of lipid vesicles during sample handling and differences in the interaction of BSA and AqpZ with the Coomassie blue dye.

#### Calculation of permeability per AqpZ monomer (P_a_)

Based on the successful incorporation of 66 ± 4% of AqpZ added, the number of AqpZ tetramer per vesicle is 35 ± 2 and P_a_ is 47 × 10^−14^ cm^3^/s/subunit. With 95% confidence interval, the average P_a_ is between 41 × 10^−14^ cm^3^/s/subunit to 51 × 10^−14^ cm^3^/s/subunit. Once again, this shows the applicability of the quantification results to determine P_a_, for evaluation of AqpZ performance.

Gel electrophoresis is a quantitative method for determining AqpZ reconstituted into vesicles. While it is difficult to detect a small percentage of AqpZ which are not reconstituted as compared to ICP-MS method, it requires smaller sample consumption and a shorter duration to complete.

### Comparison of quantification methods

The three methods presented differ in terms of accuracy, sample consumption, duration, and equipment requirements. Nanogold labeling is a simple and quick method that requires the least amount of sample. However, the huge discrepancy between the number of AqpZ per vesicle determined by TEM method as compared to ICPMS and gel electrophoresis method also shows that TEM is not an accurate method to determine the number of AqpZ incorporated. The TEM method serves only as a quick check to determine if AqpZ has been reconstituted, but not the exact number. All three methods provide information about the success of reconstitution, however, further information, such as the exact number of tetramers per vesicles and percentage loss of AqpZ to reconstitution and ultracentrifugation can only be evaluated through the ICP-MS or gel electrophoresis method.

Nickel labeling and ICP-MS requires the longest time and greatest sample consumption due to the additional nickel labeling step and ICP-MS procedure. Nevertheless, it is the most sensitive method that is able to detect the small percentage of non-reconstituted AqpZ. Gel electrophoresis is a method that requires moderate time and sample amount as compared to the other two. Although the method is not sensitive towards a small percentage of non-reconstituted AqpZ, it is still able to provide a quantitative result that is comparable to that of ICP-MS. Inevitably, both methods are susceptible to systematic error arising from ultracentrifugation, loss during sample handling, transfer and labeling. As discussed previously, the measured amount of pelleted protein is likely to be the maximum amount of protein that is functionally integrated into the membrane since the pellet from ultracentrifugation may also contain non-functional integrated protein. In the case of Ni labeling, the nickel might dissociate from the His-tag during sample manipulation and ultracentrifugation. Although dissociation during dialysis is unlikely based on the low Ni concentration present in the used dialysis buffer (please refer to the supplementary data), we are unable to verify the dissociation and loss during ultracentrifugation. On the other hand, the choice of BSA as a standard and control protein may also result in protein estimation error due to the inherent difference between BSA and AqpZ and hence the interaction with Coomassie blue stain and separation performance under ultracentrifugation. Despite the limited value of BSA as a good control and standard protein, there is a lack of a better alternative. Nonetheless, these methods still allow the more accurate relationship to be drawn between water transport and AqpZ incorporation.

The three quantification methods as presented above offer common advantages over existing quantification methods by keeping sample handling and data processing to a minimal, while avoiding the need for uncommon equipment. This is to ensure that the quantification methods can be easily implemented in any setting to obtain information of actual Aqp reconstituted. The information obtained from the quantification methods allows a critical evaluation of vesicle performance when pieced together with SFLS results. The nanogold labeling method allows the distribution of AqpZ on vesicles to be easily observed without the ambiguity faced by the FF-TEM method. Compared to FCS and SAXS method, ICP-MS and gel electrophoresis offer a more direct read out of AqpZ reconstituted, rather than relying on the indirect observation of phenomena such as diffusion time and curvature of vesicles. This reduced dependence on uncommon equipment, modeling and fitting, so that these methods can be easily implemented. Importantly, the three methods can be applied as a stand-alone method or applied together for verification of results. The information harnessed from different methods can also be combined to gain a holistic analysis. As demonstrated previously, TEM provides the dimensions of vesicles for the accurate derivation of AqpZ per vesicle. On the other hand, the amount of AqpZ and percentage AqpZ reconstituted obtained from ICP-MS and gel electrophoresis can be compared for verification.

## Conclusions

Three different methods to determine AqpZ reconstitution into vesicles for fabrication of biomimetic membrane have been presented. With the current method of reconstitution, the percentage of AqpZ successfully reconstituted can be determined. It was shown that the results obtained using different methods are comparable and correlate well to vesicle permeability. Furthermore, the quantification methods also provide insights on the predominant cause of reduced reconstitution, allowing better optimization strategies to be designed. Most importantly, the methods offer several advantages, without the need for uncommon equipment, complex sample handling, and data analysis. It is anticipated that these methods can facilitate an accurate understanding of water transport in relation to the protein reconstitution and also optimization of conditions for vesicle preparation. These would enable the fabrication of biomimetic membranes with better and well-controlled performance.

## Materials and Methods

### Materials

*E. coli* lipid was purchased from Avanti Polar Lipids. Dialysis cassettes and zebra desalting columns were purchased from Thermo Fischer Scientific. 5 nm nickel-nitrilotriacetic acid (Ni-NTA) nanogold was purchased from Nanoprobes. Gel electrophoresis reagents and apparatus were purchased from Bio-Rad. All other chemicals and solvents were purchased from Sigma-Aldrich unless otherwise mentioned.

### Expression and purification of Aquaporin-Z

The modified pET plasmid containing DNA sequence encoding AqpZ with N-terminal 6× his affinity tag was transformed into *E. coli* strain BL21 Star (DE3) (Invitrogen, Carlsbad, CA). Cells from a single colony were inoculated in LB medium with 100 µg/ml ampicillin and grown overnight at 37 °C. The overnight cultures were diluted 100-fold into fresh LB medium and propagated to an A_600 nm_ of 1.2–1.5. The culture was induced with 1 mM isopropyl b-D-1-thiogalactopyranoside and grown at 37 °C for 2 h before harvesting.

The cells of 1 L culture was harvest by centrifugation at 6000 rpm for 15 min and resuspended in 10 mL of lysis buffer containing 20 mM Tris–HCl (pH 8.0), 100 mM NaCl, 1 mM MgSO_4_, 1 mM phenylmethanesulfonyl fluoride and 0.1 mg/mL deoxyribonuclease I. Cell resuspension was subjected to sonication and the lysate was centrifuged at 10,000 g for 30 min to remove the insoluble material. The membrane fraction was recovered from the supernatant by centrifugation at 140, 000 g for 1 h.

For AqpZ extraction, the membrane fraction was resuspended in solubilization buffer (1% n-dodecyl-beta-maltoside (DDM) in a buffer containing 20 mM Tris–HCl (pH 8.0), 100 mM NaCl) and incubated overnight at 4 °C. Insoluble material was pelleted by 45 min centrifugation at 140, 000 g. The DDM solubilized AqpZ was bound to cobalt resin by gentle shaking at 4 °C for 2 h in the presence of 5 mM imidazole. The protein bound cobalt resin was washed with 10 column volumes of buffer containing 20 mM Tris–HCl (pH 8.0), 100 mM NaCl, 10 mM imidazole and 0.2% DDM. AqpZ was then eluted with washing buffer supplemented with 150 mM imidazole.

### *E. coli* vesicles preparation and AqpZ reconstitution

*E. coli* lipid vesicles were prepared using dialysis incorporation method according to a previously reported protocol^3^. Briefly, *E. coli* lipids were dissolved in chloroform and dried into a thin film under vacuum. The thin film was rehydrated in 0.1 M MOPS buffer, adjusted to pH 7.4 using 10 M sodium hydroxide. Lipid was mixed with AqpZ and 1.25% (wt/vol) of octyl glucoside (OG) detergent with buffer until homogeneous. Lipid to AqpZ weight ratio was kept at 100. The mixture was then transferred into a 10 kDa molecular weight cut-off dialysis cassette for dialysis against 0.1 M MOPS-Na buffer over three days. During the process, dialysis buffer was replaced with fresh buffer daily.

### Vesicle characterization

Vesicle size was measured via dynamic light scattering (Zetasizer Nano ZSP equipped with Helium-Neon laser beam at 633 nm, Malvern Instrument Ltd., Malvern, UK). Three measurements were made to obtain an average value for each reading.

Vesicle permeability was studied using stopped-flow apparatus spectrometer (Applied Photophysics, UK) method. Vesicle solution was quickly mixed with sucrose buffer at 0.6 osmol/L concentration. The high osmolarity of sucrose will result in water efflux from vesicles, which was recorded in the form of increasing light scattering signal. The initial gradient of the signal curve can be fitted to an exponential equation to represent the rate of change in vesicle shrinkage. The osmotic water permeability was then calculated using following equation (5).

5 $${{\rm{P}}}_{{\rm{f}}}=\frac{{\rm{k}}}{\frac{{{\rm{S}}}_{{\rm{A}}}}{{{\rm{V}}}_{{\rm{0}}}}}\times {{\rm{V}}}_{{\rm{w}}}\times {{\rm{\Delta }}}_{{\rm{osm}}}$$

where P_f_ is the osmotic water permeability (m/s), S_A_ is the vesicle surface area (m^2^), V_0_ is the initial vesicle volume (m^3^), V_w_ is the partial molar volume of water (0.018 L/mol) and Δ_osm_ is the osmolarity difference that drives the vesicle shrinkage (osmol/L).

The permeability per AqpZ subunit (P_a_) was determined using the method as described in another work^3^, using equation (6).

6 $${{\rm{P}}}_{{\rm{a}}}=\frac{{({\rm{P}}}_{{\rm{f}},{\rm{z}}}-{{\rm{P}}}_{{\rm{f}},{\rm{c}}})\times {\rm{S}}}{{\rm{m}}}$$

where P_f,z_ and P_f,c_ is the osmotic water permeability (m/s) of AqpZ incorporated and control vesicles respectively, S is the vesicle surface area (m^2^), and m is the number of AqpZ monomer subunit incorporated into vesicles.

The vesicle molecular weight was calculated using the following equation (7)^26^.

7 $${\rm{M}}=\frac{{4{\rm{\pi }}{\rm{{\rm N}}}}_{{\rm{A}}}}{{\rm{3v}}}({{\rm{3R}}}^{{\rm{2}}}{\rm{\delta }}-{\mathrm{3R}{\rm{\delta }}}^{{\rm{2}}}+{{\rm{\delta }}}^{{\rm{3}}})$$

The equation is based on interpolation of Zimm plot, an approximation of static light scattering from macromolecular solution whereby intermolecular interactions only occur through a single contact^26^. This assumption is valid for our vesicle samples in this case which are dilute macromolecular sample. In this equation, R is the vesicles radius, δ is the membrane thickness of the vesicles, N_A_ is the Avogadro’s number and v is the specific volume of the vesicle. v is assumed to be 1 cm^3^/g, as reported previously^3^. The loss of lipid vesicles during the measurements was assumed to be negligible.

### Ultracentrifugation

Ultracentrifugation was applied to separate non-reconstituted AqpZ from AqpZ in vesicles. Vesicle samples were centrifuged at 250, 000 g for 2 hours at 4 °C with Optima MAX XP ultracentrifuge (Beckman Coulter Inc, USA). For the first round of ultracentrifugation, 500 µL of 10 mg/mL *E. coli* vesicle sample was loaded in each vial for ultracentrifugation. After which, resultant supernatant was collected and labeled as supernatant (S) fraction. The pellet was suspended in 200 µL of 0.1 M MOPS-Na buffer at pH 7.4. The solution was then subjected to a second round of ultracentrifugation. Following, the resultant supernatant was collected and labeled as wash (W) fraction while the pellet was suspended in 200 µL of 0.1 M MOPS-Na buffer at pH 7.4 and labeled as pellet (P) fraction. The S, W, P fractions collected were subjected to further analysis using TEM, gel electrophoresis, and ICP-MS. An equal amount of vesicles from each fraction was loaded for all analysis.

### Nanogold labeling

A drop of sample solution at 0.5 mg/mL of lipid was dropped on TEM grid, followed by nanogold solution at 10 times of the calculated amount of AqpZ by molar ratio. The solutions were incubated on the grid for 20 min and excess solution was wicked off with a filter paper. A drop of blocking buffer, 150 mM imidazole in 100 mM NaCl solution, was dropped on the grid and incubated for 2 min. The grid was washed with ultrapure water and stained with 1% phosphotungstic acid solution. Excess solution was wicked off and the grid was air dried thoroughly before TEM visualization.

### Transmission electron microscopy

The samples were observed using JEM-2100F Transmission electron microscope (JEOL, USA) at accelerating voltage of 200 kV.

### Preparation of nickel labeled AqpZ

AqpZ or control without AqpZ was incubated with nickel chloride labeling solution (0.16 M) at room temperature, 750 rpm shaking speed for 2 h. After labeling, unbound nickel was removed with Zeba spin desalting column (40 kDa molecular weight cut off), following with dialysis (2 kDa molecular weight cut-off) against 20 mM Tris-HCl buffer (pH 7.4) containing 100 mM NaCl with 0.2% DDM, at 200 times of sample volume over four days. During the process, dialysis buffer was replaced daily.

### Sodium dodecyl sulfate polyacrylamide (SDS-PAGE) gel electrophoresis

An aliquot of the samples was incubated for 0.5 h at room temperature in sample loading buffer containing 1% sodium dodecyl sulfate (SDS) and then analyzed by SDS-PAGE of 15% polyacrylamide gels with further staining by Coomassie brilliant blue G-250. The stained gels were scanned with G BOX gel doc system from Syngene and analyzed with the software of GeneTools to identify the protein size and amount.

### Inductively coupled plasma-mass spectrometry

The sample was firstly digested with Ethos One microwave digester (Milestone Inc, USA). Measurement of nickel concentration of digested sample was then made on Agilent 7700x ICP-MS system. The operating conditions are summarized in Table 4. In all experiments, the sample uptake was directed through Agilent integrated autosampler with a micro mist nebulizer. Calibration samples were prepared from dilution of Periodic table mix 1 for ICP purchased from Sigma Aldrich.

**Table 4:** ICP-MS operating conditions (Agilent 7700x ICP-MS).

Parameter	Description
Sample introduction	Peristaltic pump
	Agilent integrated autosampler (I-AS)
	Micromist nebulizer (0.10 rps)
RF plasma source power	1550 W
Plasma gas	15.02 L/min
Auxiliary gas	0.90 L/min
Carrier gas	1.03 L/min

## Electronic supplementary material

Supplementary Information

## References

[1] Shannon MA (2008). Science and technology for water purification in the coming decades. Nature.

[2] Fane AG, Wang R, Hu MX (2015). Synthetic membranes for water purification: status and future. Angewandte Chemie International Edition.

[3] Borgnia MJ, Kozono D, Calamita G, Maloney PC, Agre P (1999). Functional reconstitution and characterization of AqpZ, the E. coli water channel protein. Journal of molecular biology.

[4] Lee KP, Arnot TC, Mattia D (2011). A review of reverse osmosis membrane materials for desalination—Development to date and future potential. Journal of Membrane Science.

[5] Pendergast MM, Hoek EMV (2011). A review of water treatment membrane nanotechnologies. Energy & Environmental Science.

[6] Li X (2012). Preparation of supported lipid membranes for aquaporin Z incorporation. Colloids and Surfaces B: Biointerfaces.

[7] Zhao Y (2012). Synthesis of robust and high-performance aquaporin-based biomimetic membranes by interfacial polymerization-membrane preparation and RO performance characterization. Journal of Membrane Science.

[8] Sun G, Chung T-S, Jeyaseelan K, Armugam A (2013). A layer-by-layer self-assembly approach to developing an aquaporin-embedded mixed matrix membrane. RSC Advances.

[9] Sun G, Chung T-S, Jeyaseelan K, Armugam A (2013). Stabilization and immobilization of aquaporin reconstituted lipid vesicles for water purification. Colloids and Surfaces B: Biointerfaces.

[10] Li X (2014). Preparation of high performance nanofiltration (NF) membranes incorporated with aquaporin Z. Journal of Membrane Science.

[11] Sun G, Chung T-S, Chen N, Lu X, Zhao Q (2013). Highly permeable aquaporin-embedded biomimetic membranes featuring a magnetic-aided approach. RSC Advances.

[12] Wang H (2012). Highly Permeable and Selective Pore‐Spanning Biomimetic Membrane Embedded with Aquaporin Z. Small.

[13] Wang HL (2013). Mechanically robust and highly permeable AquaporinZ biomimetic membranes. Journal of Membrane Science.

[14] Zhong PS, Chung T-S, Jeyaseelan K, Armugam A (2012). Aquaporin-embedded biomimetic membranes for nanofiltration. Journal of Membrane Science.

[15] Duong PH (2012). Planar biomimetic aquaporin-incorporated triblock copolymer membranes on porous alumina supports for nanofiltration. Journal of Membrane Science.

[16] Qi S (2016). Aquaporin-based biomimetic reverse osmosis membranes: Stability and long term performance. Journal of Membrane Science.

[17] Wang M (2015). Layer-by-layer assembly of aquaporin Z-incorporated biomimetic membranes for water purification. Environmental science & technology.

[18] Xie W (2013). An aquaporin-based vesicle-embedded polymeric membrane for low energy water filtration. Journal of Materials Chemistry A.

[19] Habel J (2015). Aquaporin-Based Biomimetic Polymeric Membranes: Approaches and Challenges. Membranes (Basel).

[20] Grzelakowski M, Cherenet MF, Shen Y-X, Kumar MA (2015). framework for accurate evaluation of the promise of aquaporin based biomimetic membranes. Journal of Membrane Science.

[21] Valenti LE, De Pauli CP, Giacomelli CE (2006). The binding of Ni(II) ions to hexahistidine as a model system of the interaction between nickel and His-tagged proteins. Journal of Inorganic Biochemistry.

[22] Bornhorst JA, Falke JJ (2000). [16] Purification of Proteins Using Polyhistidine Affinity Tags. Methods in enzymology.

[23] Ersöz A, Say R, Denizli A (2004). Ni(II) ion-imprinted solid-phase extraction and preconcentration in aqueous solutions by packed-bed columns. Analytica Chimica Acta.

[24] Figueroa, J. A. L., Stiner, C. A., Radzyukevich, T. L. & Heiny, J. A. Metal ion transport quantified by ICP-MS in intact cells. *Scientific reports* **6** (2016). 10.1038/srep20551 PMC4738345 26838181

[25] Nieba L (1997). BIACORE analysis of histidine-tagged proteins using a chelating NTA sensor chip. Analytical biochemistry.

[26] van Zanten JH (1994). Unilamellar Vesicle Diameter and Wall Thickness Determined by Zimm’s Light Scattering Technique. Langmuir.

[27] Hashido M, Ikeguchi M, Kidera A (2005). Comparative simulations of aquaporin family: AQP1, AQPZ, AQP0 and GlpF. FEBS letters.

[28] Borgnia MJ, Agre P (2001). Reconstitution and functional comparison of purified GlpF and AqpZ, the glycerol and water channels from Escherichia coli. Proceedings of the National Academy of Sciences.

[29] Scheuring S (1999). High resolution AFM topographs of the Escherichia coli water channel aquaporin Z. The EMBO Journal.

[30] Congdon RW, Muth GW, A. G. S (1993). The Binding Interaction of Coomassie Blue with Proteins. Anal Biochem..

[31] Kozono D (2003). Functional expression and characterization of an archaeal aquaporin AqpM from Methanothermobacter marburgensis. Journal of Biological Chemistry.

