# Supplementary material for: Quantification of Aquaporin-Z reconstituted into vesicles for biomimetic membrane fabrication
Source: Sci Rep. 2017 Sep 14;7:11565. doi: 10.1038/s41598-017-11723-x (PMC5599656; doi:10.1038/s41598-017-11723-x)

**Quantification of Aquaporin-Z reconstituted into vesicles for biomimetic membrane fabrication**

**Hui Xian Gan**^1, 2^, ^+^, **Hu Zhou**^1, 3, +^, **Qingsong Lin**^1, 3^, * and **Yen Wah Tong**^1, 2^, *

^1^ National University of Singapore, NUS Environmental Research Institute (NERI), Singapore, 117411, Singapore

^2^ National University of Singapore, Chemical and Biomolecular Engineering, Singapore, 117576, Singapore

^3^ National University of Singapore, Department of Biological Sciences, Singapore, 117543, Singapore

*Correspondence: Yen Wah Tong: chetyw@nus.edu.sg; Qingsong Lin: dbslinq@nus.edu.sg

^+^These authors contributed equally to the work.

Supplementary information

| NiCl_2_ concentration (mM) | Removal efficiency (%) |
| --- | --- |
| 10 | 99.8690 |
| 25 | 99.9521 |
| 50 | 99.9658 |

**Table S1. Nickel removal efficiency using 2k MWCO dialysis cassette for control buffer labelled with different nickel chloride concentration.**

Note: $\text{Removal efficiency = }100\times\frac{\text{Concentration after Ni removal}}{\text{Concentration before Ni removal}}$

| NiCl_2_ concentration (mM) | Removal efficiency (%) |
| --- | --- |
| 10 | 99.9836 |
| 25 | 99.9934 |
| 50 | 99.9964 |

**Table S2. Nickel removal efficiency using 25k MWCO dialysis cassette for control buffer labelled with different nickel chloride concentration.**

Note:

| NiCl_2_ concentration (mM) | Removal efficiency (%) |
| --- | --- |
| 10 | 99.9786 |
| 25 | 99.9934 |
| 50 | 99.9953 |

**Table S3. Nickel removal efficiency using 50k MWCO dialysis cassette for control buffer labelled with different nickel chloride concentration.**

Note: $\text{Removal efficiency = }100\times\frac{\text{Concentration after Ni removal}}{\text{Concentration before Ni removal}}$

|  | Dilution before ICP-MS | ICP concentration (ppb) |
| --- | --- | --- |
| 0M Ni labelled OG Buffer | 100x dilution | 0.269 |
| 0.5M Ni labelled OG Buffer | 100x dilution | 148 |
| 1M Ni labelled OG Buffer | 100x dilution | 247 |

**Table S4. Measured nickel concentration of labelled samples before nickel removal.** Note: Dilution is due to microwave digestion.

|  | Dilution before ICP-MS | ICP concentration (ppb) | Removal efficiency (%) |
| --- | --- | --- | --- |
| 0M Ni labelled OG Buffer | 100x dilution | 0.278 | - |
| 0.5M Ni labelled OG Buffer | 100x dilution | 16.2 | 89.0541 |
| 1M Ni labelled OG Buffer | 100x dilution | 20.9 | 91.5385 |

**Table S5. Measured nickel concentration of labelled samples after using 40k MWCO spin column for nickel removal.** Note: Dilution is due to microwave digestion.

|  | Dilution before ICP-MS | ICP concentration (ppb) | | Removal efficiency (%) |
| --- | --- | --- | --- | --- |
| 0M Ni labelled OG Buffer | 0x dilution | | 0.481 | - |
| 0.5M Ni labelled OG Buffer | 0x dilution | | 0.312 | 99.9979 |
| 1M Ni labelled OG Buffer | 0x dilution | | 0.462 | 99.9981 |

**Table S6. Measured nickel concentration of labelled samples after 40k MWCO spin column removal and dialysis using 2k MWCO dialysis cassette.** Note: Dilution is due to microwave digestion.

| Sample | ICP concentration (ppb) |
| --- | --- |
| Before use | 1.60 |
| First dialysis | 67.8 |
| Third dialysis | 1.97 |
| Fourth dialysis | 1.74 |

**Table S7. Measured nickel concentration of dialysis buffer collected after using for dialysis to remove unbound nickel from control buffer.** Note: Dialysis buffer was replaced with fresh dialysis buffer after each round of dialysis (daily) and a total of three replacements were made. First, third and fourth dialysis refers to the dialysis buffer after the first round, third round and fourth round of dialysis respectively.

**Figure S1. Gel electrophoresis results of different fractions of BSA-doped E. coli vesicles.** ECBSA refers to BSA-doped E. coli control sample. P, W and S refer to the pellet, wash and supernatant fraction obtained from after ultracentrifugation. BSA refers to BSA standard solution loaded in known mass as indicated.


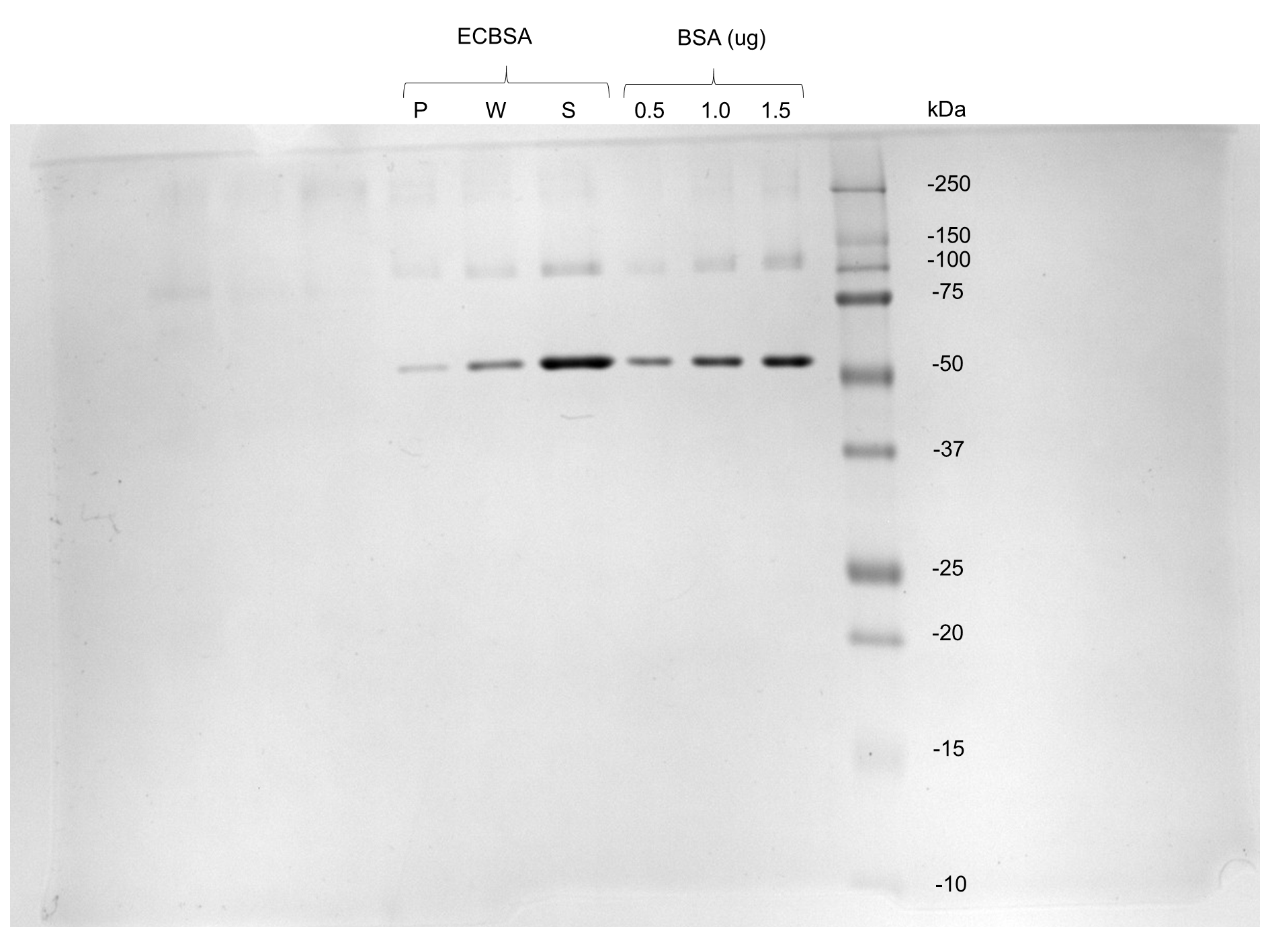


**Figure S2. Electrophoresis results of AqpZ incorporated vesicles.** (a-c) Electrophoretic analysis of E. coli lipid samples before and after incorporation via dialysis. Samples were derived from the same experiment and the gels were processed using same settings in parallel. Sample containing 1 ug of AqpZ or equivalent volume of control buffer is loaded for gel electrophoresis. Duplicates of each sample were loaded for each gel electrophoresis for (a-b). Before and after refer to before and after dialysis respectively. Pellet, wash, and supernatant refers to the different fractions collected from ultracentrifugation separation process. ECC and ECZ refer to control and AqpZ reconstituted E. coli lipid vesicles respectively. ECCNi and ECZNi refer to nickel labelled control and AqpZ reconstituted E. coli lipid vesicles respectively. NiZ refers to nickel labelled AqpZ and Z refers to non-nickel labelled AqpZ.


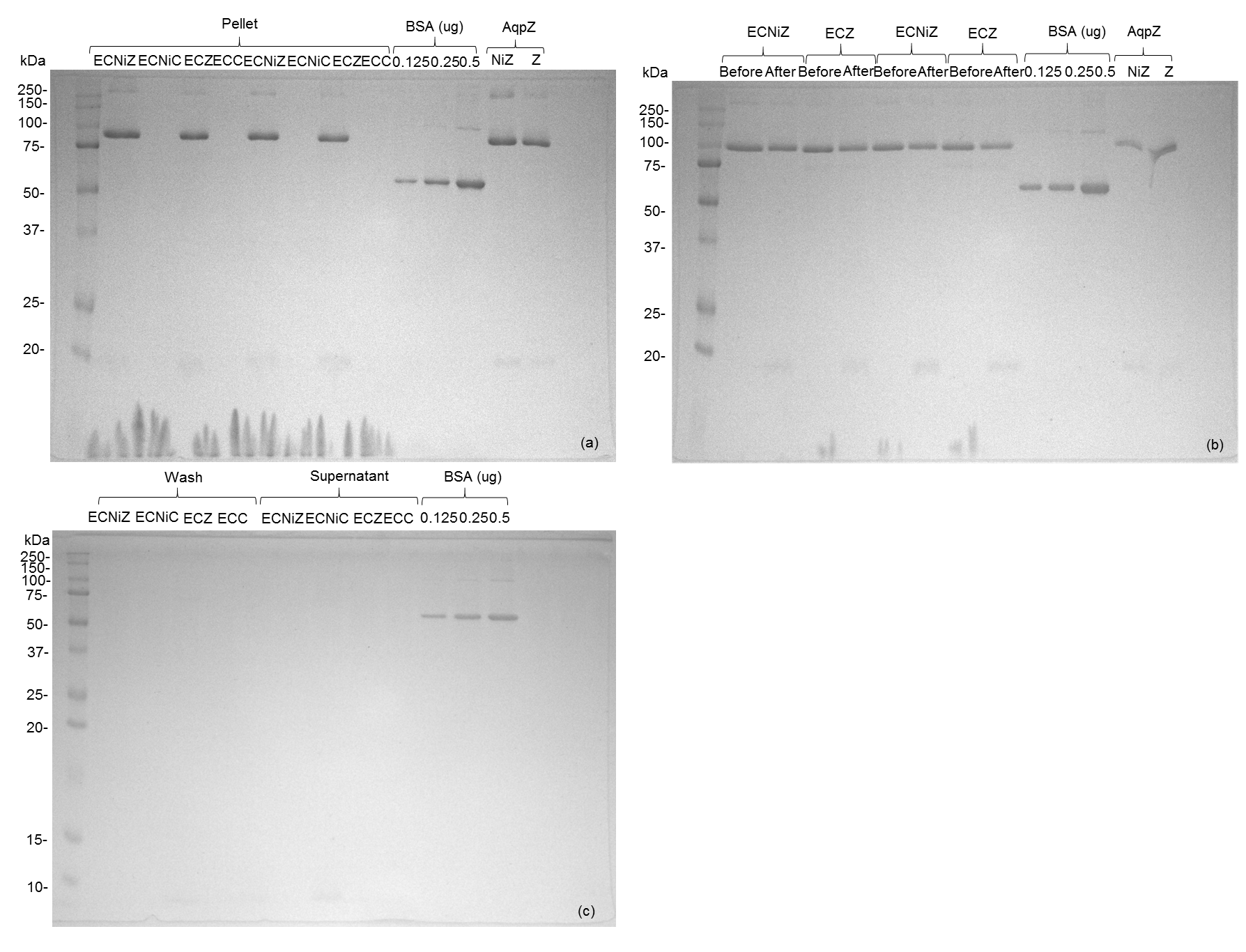

Supplement: Supplementary file 1 — Supplementary Information [file 41598_2017_11723_MOESM1_ESM.doc]
